# Evaluating the accuracy of a cataract surgery simulation video in depicting patient experiences under conscious anesthesia

**DOI:** 10.1007/s10792-023-02892-y

**Published:** 2023-10-24

**Authors:** Ruti Sella, Rebecca R. Lian, Anser A. Abbas, Spencer D. Fuller, Sean S. Bentley, Hideki Fukuoka, Natalie A. Afshari

**Affiliations:** Shiley Eye Institute, Viterbi Family Department of Ophthalmology, University of California San Diego, 9415 Campus Point Dr #0946, La Jolla, CA 92093-0946 USA

**Keywords:** Cataract surgery, Patient experience, Simulation video, Visual experience, Anxiety, Pain

## Abstract

**Purpose:**

To evaluate the accuracy of a point-of-view cataract surgery simulation video in representing different subjective experiences of patients undergoing the procedure.

**Methods:**

One hundred consecutive post-cataract-surgery patients were shown a short simulation video of the surgery obtained through a porcine eye model during the first postoperative week. Patients then answered a multiple-choice questionnaire regarding their visual and tactile intraoperative experiences and how those experiences matched the simulation.

**Results:**

Of the patients surveyed (*n* = 100), 78% (*n* = 78) recalled visual experiences during surgery, 11% recalled pain (*n* = 11), and 6.4% (*n* = 5) recalled frightening experiences. Thirty-six percent of patients (*n* = 36) were interviewed after their second cataract surgery; there was no statistically significant difference between anxiety scores reported before the first eye surgery and second eye surgery (*p* = 0.147). Among all patients who recalled visual experiences (*n* = 78), nearly half (47.4%) reported that the video was the same/similar to their experience. Forty-eight percent of the patients recommended future patients to watch the video before their procedures, and more than a third (36%) agreed that watching the video before surgery would have helped them to relax.

**Conclusions:**

Our model reflects the wide range of subjective patient experiences during and after surgery. The high percentage of patients who found the video accurate in different ways suggests that, with more development, point-of-view cataract simulation videos could prove useful for educational or clinical use. Further research may be done to confirm the simulation’s utility, by screening the video for subjects before operations.

**Supplementary Information:**

The online version contains supplementary material available at 10.1007/s10792-023-02892-y.

## Introduction

Patients undergoing cataract surgery have reported a wide variety of visual and tactile experiences during their procedures [1–3]. Many patients have reported seeing bright lights, colors, and more rarely, surgeon’s hands with surgical instrument visualization [3–8]. Other patients have not recalled any visual phenomena altogether [3]. Patients’ experiences have been shown to differ depending on a number of factors including anesthesia route and preoperative counseling [4, 7–10].

Although the majority of patients do not find their experiences during cataract surgery distressing, it has been reported that between 3% and 19.4% of patients are frightened by visual experiences (8). In addition to intraoperative fear, patients also experience a range of anxiety before undergoing surgery [1, 2, 16]. Fear and anxiety have the potential to cause significant distress to patients, decreasing patient satisfaction. Additionally, in one study severe preoperative anxiety was associated with higher levels of intraoperative pai [1]. Furthermore, fear has the potential to result in a number of suboptimal intraoperative events including hypertension, tachycardia, ischemia, panic attack, and decreased patient cooperation during surgery, possibly leading to increased morbidity and intraoperative complications [17, 18].

Previously, it has been reported that patients who received counseling regarding potential intraoperative visual experiences during cataract surgery report significantly less fear during surgery [9]. Additionally, a number of models for reproducing patient visual perceptions during cataract surgery such as patient generated representations [19], and model eye video clips [20], have been found to be effective preoperative communication tools.

A variety of depictions of patient visual experience during cataract surgery have been indeed created for both clinician and patient education. Illustrations have been created based on patient descriptions [19, 21]; a model eye was suggested to create short video clips of different portions of cataract surgery from the patient’s point of view. [20]

Previously, our group has published a point-of-view simulation of cataract surgery created by using a porcine eye model. [22] The surgeries were video captured from a patient’s perspective (supplement video). This current study aims to examine the range of subjective experiences of patients undergoing cataract surgery and to assess the ability of the aforementioned video clips to represent visual phenomena experienced by patients.

## Methods

Informed consent was obtained from each patient before enrollment in the study. Human resource protection program approval was obtained. The described research adhered to the tenets of the Declaration of Helsinki.

A short video simulating the experience of cataract surgery from the patient’s point of view was created by filming a full cataract surgery from a 3 mm maculostomy through the posterior globe of a porcine eye. Full details regarding the methods of creating this video were previously published. (supplementary information) [22]

In this prospective study, 100 consecutive postoperative patients who had undergone cataract surgery were shown the above-described point-of-view cataract surgery simulation video at follow-up appointments within one week of cataract surgery. All eyes included in the study received cataract surgery under topical anesthesia with monitored anesthesia care (MAC) anesthesia at the same academic center. Table 1 demonstrates the type, dosage, and route of sedation received. by patients in our study.

**Table 1:** Range of sedation regimens in operated patients under monitored anesthesia care (MAC,* n* = 100)

Sedation regimen used	Midazolam (1–4 mg IV)^*^ and Fentanyl (25–100mcg IV)^*^	Midazolam (1–4 mg IV)^*^ and Propofol (10 mg, IV)^*^	Fentanyl only (25–100mcg IV)^*^	No sedation
% of patients	95%	1%	3%	1%

*IV (intravitreal) dose at the discretion of the anesthesiologist

Sedative dose was adjusted by anesthesiologist based on body mass index and comorbidities at the discretion of the anesthesiologist. Surgery was performed by standard phacoemulsification technique with a foldable intraocular lens placement. Excluded were patients younger than 18 years old, or patients with postoperative best-corrected visual acuity worse than 20/50 in both eyes, rendering them unable to perceive the video visually.

After watching the simulation video, patients were given a short multiple-choice questionnaire. The survey included questions regarding physical and visual experiences during surgery, as well as evaluation of the eye simulation video. Table 2 includes all questions given to patients in the survey.

**Table 2:** Survey questions answered by postoperative cataract patients after watching animal model cataract surgery simulation video

Question	*N* (total)	% (total)	*N* (unilateral patients)	% (unilateral patients)	*N* (bilateral patients)	% (bilateral patients)
Did you recall any awareness of visual experiences during your own surgery?						
Yes	78	78	49	76.60	29	80.60
No	22	22	15	23.40	7	19.40
If you answered yes to question 1, how similar to the video was the visual experience during your own cataract surgery?						
1. Same	3	3.80	1	2.00	2	6.90
2. Similar	34	43.60	21	42.90	13	44.80
3. Different	38	48.70	25	51.00	13	44.80
4. Do Not Know	3	3.80	2	4.10	1	3.40
Do you recall any bright lights, flashes, or other light intensity changes during your surgery?						
Yes	35	65	41	64.10	24	66.70
No	35	35	23	35.90	12	33.30
If you answered yes to question 3, how similar to the video was the experience with light intensity changes during your procedure?						
1. Same	10	15.40	5	12.20	5	20.80
2. Similar	20	30.80	12	29.30	8	33.30
3. Different	32	49.20	21	51.20	11	45.80
4. Do Not Know	3	4.60	3	7.30	0	3.40
Do you recall any instrument visualization or other object awareness during your procedure?						
Yes	18	18	8	12.50	10	27.80
No	82	82	56	87.50	26	72.20
If you answered yes to question 5, how similar to the video was the experience with instrument visualization and object awareness during your procedure?						
1. Same	4	22.20	1	12.50	3	30.00
2. Similar	9	50.00	4	50	5	50.00
3. Different	4	22.20	2	25	2	20.00
4. Do Not Know	1	5.60	1	12.50	0	0.00
Do you recall any frightening visual experiences during your surgery?						
Yes	5	5	3	4.70	2	5.60
No	95	95	61	95.30	34	94.40
Do you recall any pain during your surgery?						
Yes	11	11.00	7	10.90	4	11.10
No	89	89.00	57	89.10	32	88.90
If you answered yes to question 8, please rate your level of pain on a scale of 1 to 10. (10 being most painful)						
1	1	9.10	0	0.00	1	25.00
2	4	36.40	2	28.60	2	50.00
3	1	9.10	1	14.30	0	0.00
4	2	18.20	2	28.60	0	0.00
5	2	18.25	1	14.30	1	25.00
6	1	9.10	1	14.30	0	0.00
7	0	0.00	0	0.00	0	0.00
8	0	0.00	0	0.00	0	0.00
9	0	0.00	0	0.00	0	0.00
10	0	0.00	0	0.00	0	0.00
N/A	89	NA	57	NA	32	NA
After watching this video, did your level of anxiety about your cataract surgery:						
Increase	13	13.00	11	17.20	2	5.70
Decrease	16	16.00	11	17.20	5	14.30
Remain the Same	71	71.00	42	65.60	29	80.00
Do you think that watching the video before surgery would have helped to relax you?						
1. Not at all	48	48.00	31	48.40	17	47.20
2. Not so much	14	14.00	8	12.50	6	16.70
3. I agree so	27	27.00	19	29.70	8	22.20
4. I strongly agree so	9	9.00	5	7.80	4	11.10
5. Do not know	2	2.00	1	1.60	1	2.80
Do you recommend other patients to watch the simulation video before they have cataract surgery?						
Yes	48	48.00	30	47.60	17	47.20
No	52	52.00	33	52.40	19	52.80
If this is your second cataract eye surgery:						
(a)How was your level of anxiety before the 1^st^ procedure? On a scale of 1 to 10 (10 being most anxious)						
1	9	25.70	NA	NA	9	25.70
2	2	5.70	NA	NA	2	5.70
3	5	14.30	NA	NA	5	14.30
4	2	5.70	NA	NA	2	5.70
5	6	17.10	NA	NA	6	17.10
6	3	8.60	NA	NA	3	8.60
7	1	2.90	NA	NA	1	2.90
8	3	8.60	NA	NA	3	8.60
9	2	5.70	NA	NA	2	5.70
10	2	5.70	NA	NA	2	5.70
N/A	65	NA	NA	NA	65	NA
(b)How was your level of anxiety before the 2nd procedure? On a scale of 1–10 (10 being most anxious)						
1	11	31.40	NA	NA	11	31.40
2	3	8.60	NA	NA	3	8.60
3	6	17.10	NA	NA	6	17.10
4	0	0.00	NA	NA	0	0.00
5	4	11.40	NA	NA	4	11.40
6	1	2.90	NA	NA	1	2.90
7	4	11.40	NA	NA	4	11.40
8	1	2.90	NA	NA	1	2.90
9	0	0.00	NA	NA	0	0.00
10	2	5.70	NA	NA	2	5.70
N/A	65	NA	NA	NA	65	NA

Based on the survey results, the proportion of patients who reported various visual and tactile experiences were calculated. Among the patients who recalled a given visual element (For example: lights and flashes, or instrument visualization) during cataract surgery, the proportion of patients who reported that the simulation video was the same or similar to their experience was calculated. Among patients who reported any intraoperative pain, their pain was measured on a numerical rating scale of 1 to 10 [23], with one being low pain and 10 being extreme pain.

Among patients who had undergone delayed sequential bilateral cataract surgeries, preoperative anxiety was measured with a 10-point modified Likert scale (range:1–10, 1 = lowest anxiety, 10 = extreme anxiety) [24]. The average anxiety score was evaluated for both the first and second cataract surgery. Differences between preoperative anxiety scores were determined using the Wilcoxon signed-rank test. The percentage of patients who reported greater, equal, and less anxiety before their first eye surgery compared to their second eye surgery were also calculated.

Respondents were then divided into two groups: those who did recommend the video simulation and those who did not. Proportions of patients who had reported various experiences during cataract surgery were calculated within each subgroup. Two sample *Z* tests were used to analyze differences between subgroups. Differences were considered significant if the *P* value was < 0.05. Calculated means and standard deviations were expressed as mean ± SD.

Potential differences between the unilateral patient and bilateral patient subgroups were evaluated using a Chi-squared test, and differences were considered significant if the *P* value was < 0.05.

## Results

There were 100 patients surveyed in this study. Patients surveyed were 55% female and 45% male. In this study, 65% of those patients received only 1% intracameral lidocaine as their topical anesthesia, while 32% received both 1% intracameral lidocaine and 2% topical lidocaine jell. Only 3% of patients received 1% Intracameral lidocaine and 0.5% topical tetracaine during surgery.

Of the patients surveyed (*n* = 100), 78.8% (*n* = 78) recalled visual experiences during surgery. Among those who recalled visual experiences (*n* = 78), 83.3% reported seeing lights/flashes, 23.1% recalled seeing objects/instruments, and 6.4% (*n* = 5) recalled frightening experiences.

**Fig. 1:**
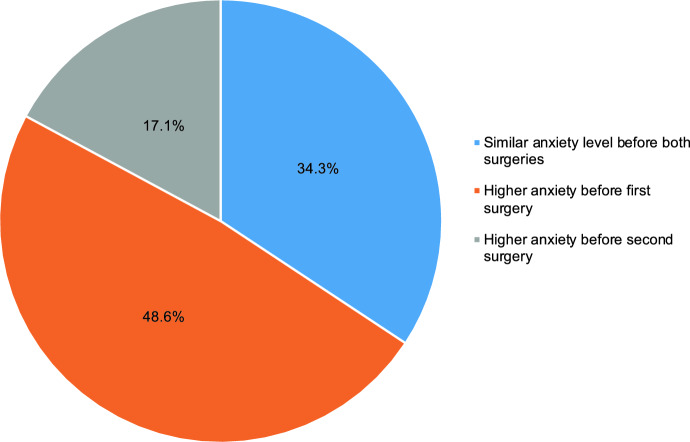
Patient self-reported anxiety levels before first and second eye cataract surgeries

Of the total cohort, 36% (*n* = 36) were interviewed after their second cataract surgery. Among these patients, there was no significant difference between average anxiety reported before first surgery and the second surgery, with average anxiety changing by − 0.77 (95% CI: 0.38, − 1.93) between the first and second procedure (*p* = 0.147). More anxiety before the first eye surgery was reported in 48.6% of patients, while higher anxiety before the second eye surgeries was reported by 17.1% of patients, and 34.3% of patients reported the same level of anxiety before both surgeries (Fig. 1). When evaluating the video’s accuracy, there were no statistically significant differences between patients interviewed after their second surgery, and the first. However, 17.2% (*n* = 11) of unilateral patients reported feeling more anxiety after watching the video versus 5.7% (*n* = 2) of bilateral patients.

Of the 100 included patients, 11% recalled pain during surgery (*n* = 11). The average grading of pain (on a scale of 1–10) reported by those who recalled pain (*n* = 11) was 3.3 ± 1.6. Among those who recalled visual experiences (*n* = 78), 47.4% reported that the video was the same/similar to their experience. Of those who recalled lights/flashes (*n* = 65), 46.2% reported that the simulation was the same/similar to that aspect of their surgery. Of those who recalled instrument visualization (*n* = 18), 72.2% reported that the simulation was the same/similar to that aspect of their surgery. Only 36% of patients agreed that watching the video before surgery would have helped to relax them; however, 48% recommended other patients watch the video before their procedures. Five percent of patients (6.4% of those who recalled visual experiences) recalled frightening visual experiences during surgery.

## Discussion

In a previous paper, our group had presented a new tool, a point of view video using a porcine eye, for modeling patient visual experiences during cataract surgery. In this current study, we demonstrate that nearly half of the patients shown this video, found it similar to their experience. The most similar model to ours was published by Inoue et al., who surveyed 20 patients regarding video clips simulating cataract surgery using a model eye. Compared to our results, Inoue et al. reported that video clips were “the same” or “similar” to patient experiences in 50%–70% of patients. [20] An even higher percentage of patients, 80%, recommended the video clips to future patients. [20] However, compared to the 20 eyes included in the paper by Inoue et al., our study has a much larger sample size, with 100 eyes included. Additionally, our study has very significant differences in anesthesia and sedation compared to previous studies evaluating patient visual experiences during cataract surgery [4–6, 8, 11–13, 15, 17, 25–27]. In this respect, our study better represents the accuracy of our video model in representing the typical cataract surgery for patients in the USA; our study demonstrates that even under MAC anesthesia, the vast majority of patients still recall some visual experiences during surgery, and that nearly half of patients would recommend the video to future patients undergoing surgery. Because many patients considered the video to be valuable and recommended the video for future patients, we believe further research should be done to evaluate its potential for clinical use for patients who express anxiety about surgery or wonder out loud about the experience they are about to undergo.

The results of our study have also added to our knowledge regarding patient experiences during cataract surgery in general. In our study, 78% of patients surveyed reported recollection of some visual experience during surgery. This rate of visual recollection is similar to the rate reported in previous studies despite given sedation [4, 5, 5, 6, 11–13, 28], and likely represents the visual experiences of routine cataract surgery as commonly performed in the US.

Notably, in our study only 5% of the total patients surveyed (and 6.4% of those who recall any visual experiences) reported experiencing frightening visual experiences. Previous studies have reported that between 3% and 19.4% of patients experience frightening visual sensations during cataract surgeries (1–3,6, 7,9–12). Differences in the rate of fear during cataract surgery have been demonstrated between different routes of anesthesia, as well as with different preoperative counseling [12, 14]. The fact that we found that few patients reported frightening experiences during surgery suggests that sedation via MAC (Monitored anesthesia care) may reduce the incidence of patient fear without substantially altering the probability of patient recollection of visual experience.

Our study shows that only 11% of patients reported pain during this surgery. This is consistent with a previous study describing good pain control in patients undergoing cataract surgery under topical anesthesia and sedation [29], and poor pain control in the absence of sedation (11).

For patients in our study who had undergone consecutive cataract surgeries, a higher percentage of patients reported higher anxiety before the first surgery than before the second surgery. The results, however, were not statistically significant. The results of published literature on this topic have been mixed with some studies demonstrating significant differences [2, 30, 31] and others lacking statistically significant differences [31–33] between anxiety levels before first and second eye cataract surgeries. The relatively small sample of patients who had undergone two cataract surgeries in our study (*n* = 36), may limit our ability to discern subtle differences that may exist in anxiety levels between the two.

There are several limitations of our study. As discussed above, our patients received varying amounts of sedation during their cataract surgery, which could have altered or diminished their perceptions during surgery. Further research assessing this video for patients undergoing cataract surgery under topical anesthesia without sedation may give more reliable results regarding the accuracy of the representations of visual phenomena in our animal model simulation video. Additionally, there is the potential for recall bias, as patients were asked to compare the video to their experience after having undergone the surgery.

In conclusion, our eye model video accurately reflects different aspects of the visual phenomena seen by a large percentage of patients who had undergone cataract surgery. Further studies of patients undergoing surgery under local anesthesia and evaluating the utility of the video footage in patients prior to their surgery could be of importance.

### Supplementary Information

Below is the link to the electronic supplementary material.

Supplementary file 1 (MP4 52531 kb)

Supplementary file 2 (DOCX 12 KB)
