# Supplementary material for: Evaluating the accuracy of a cataract surgery simulation video in depicting patient experiences under conscious anesthesia
Source: Int Ophthalmol. 2023 Oct 24;43(12):4897–904. doi: 10.1007/s10792-023-02892-y (PMC10724339; doi:10.1007/s10792-023-02892-y)
Supplement: Supplementary file 2 — (DOCX 12 KB) [file 10792_2023_2892_MOESM2_ESM.docx]

**Supplementary Information:** Video simulation modeling a patient’s visual experience during cataract surgery. This model was created by placing the camera lens through a maculostomy in a bovine eye. Video footage was then captured through the maculostomy camera while an experienced surgeon preformed cataract surgery on the bovine eye [22].
